# Dysfunctional pulmonary artery conduit and co-existing large pseudoaneurysm: well-suited for a percutaneous approach with the Melody valve?

**DOI:** 10.1186/s40064-016-3273-3

**Published:** 2016-09-15

**Authors:** Rouven Kubicki, Brigitte Stiller, Jochen Grohmann

**Affiliations:** Department of Congenital Heart Defects and Pediatric Cardiology, Heart Center, University of Freiburg, Mathildenstraße 1, 79106 Freiburg, Germany

**Keywords:** Pseudoaneurysm, Right ventricular outflow tract conduit, Percutaneous pulmonary valve implantation, Two-stage interventional approach, Homograft degeneration

## Abstract

Pseudoaneurysm formation is a rare but potentially life-threatening complication after surgical repair of congenital heart disease. We present a boy with truncus arteriosus communis 14 years after homograft placement in pulmonary position. On follow-up, he presented progressive chronic homograft degeneration. Moreover, a large pseudoaneurysm in the right ventricular outflow tract was surprisingly depicted. We opted for a two-stage interventional approach.

**Electronic supplementary material:**

The online version of this article (doi:10.1186/s40064-016-3273-3) contains supplementary material, which is available to authorized users.

## Background

Pseudoaneurysm of the right ventricular outflow tract (RVOT) is a rare complication after surgery for congenital heart disease. A substantial number of patients are asymptomatic, however, compared to true aneurysms, pseudoaneurysms are presumed to be high risk regarding life-threatening complications due to rupture or compression of adjacent mediastinal structures. Once diagnosed, pseudoaneurysms should be treated either by surgery, or via an interventional approach (Antal et al. 2010; Levine et al. 1995; Masri et al. 2014; Sadiq et al. 1994; Yeo et al. 1998).

We report on a two-stage interventional approach for valve replacement in a degenerated right ventricular-to-pulmonary artery (RV-PA) homograft by percutaneous pulmonary valve implantation (PPVI) and exclusion of a large co-existing pseudoaneurysm.

## Case presentation

A 14-year-old boy with type A1 truncus arteriosus communis underwent Rastelli repair with a 16 mm RV-PA homograft at 6 weeks of age. He developed very well, presenting no signs of heart failure over the following years. Echocardiography showed progressive chronic homograft degeneration with moderate to severe stenosis and insufficiency. Moreover, transthoracic echocardiography unexpectedly depicted a pseudoaneurysm on follow-up (Additional file 1: Video S1). Cardiac MRI revealed a pulmonary regurgitant fraction of 50 %, end-diastolic volume of 134 ml/m^2^, dilation of the non-calcified homograft up to 18–24 mm, and a large pseudoaneurysm distal to the homograft´s valve (Additional file 2: Figure S1). This aneurysm had a short neck with a narrow ostium. Neither the patient’s history nor clinical findings suggested any infective etiology (e.g., endocarditis). Following interdisciplinary discussion, we opted for an interventional approach.

**Fig. 1:**
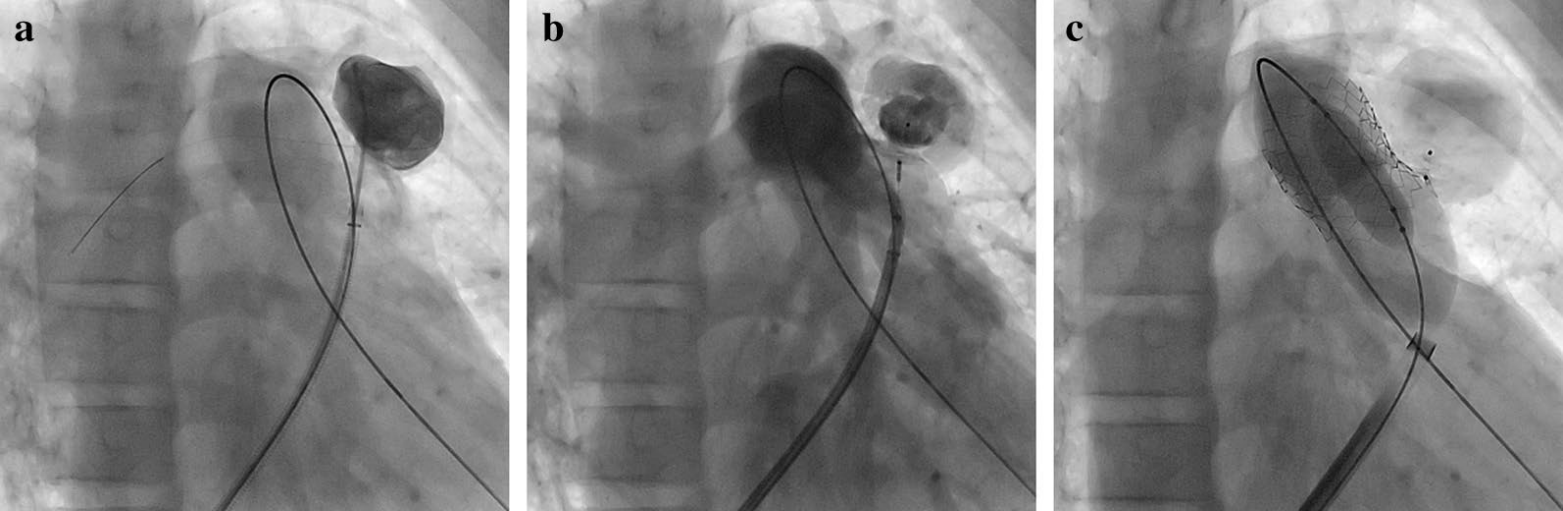
Guided by a wire-supported 8 French long sheath, the rounded-shaped pseudoaneurysm was entered via a 4 French endhole-catheter with a floppy 0.014 inch coronary wire (**a**). Its lumen and neck measured around 20 and 5 mm. Control angiography before Amplatzer™ Duct Occluder II 6–4 mm release, which was delivered via the corresponding 5 French TorqVue™ system (**b**). Implantation of the first pre-stent (**c**)

Initial hemodynamic assessments confirmed elevated right ventricular (RV) pressure up to roughly ¾ systemic (RV 92/0–12 mmHg). The RVOT angiogram revealed the nearly non-calcified homograft with an elastic appearance and its narrowest diameter measuring around 10 mm exactly on the valve’s level. The lumen of the rounded-shaped pseudoaneurysm measured around 20 mm. Its 5 mm neck was closed with an Amplatzer™ Duct Occluder (ADO II 6–4 mm; St. Jude Medical, St. Paul, MN) (Fig. 1a, b).

Next step was to create a landing zone for PPVI. Due to the compliant nature of the whole conduit, test-ballooning revealed only dynamic (muscular) proximal notching towards the RVOT, and a little notching while ballooning the distal conduit (Additional file 3: Video S2). Thus, as the pseudoaneurysm appeared effectively excluded, we decided against a long, covered stent, and chose a bare pre-stent with an open-cell design to provide sufficient anchoring (AS XXL 30 mm; Andramed, Reutlingen, Germany) crimped on a 24/40 mm Balloon-in-Balloon^®^ system (NuMed, Hopkinton, NY, USA), and implanted it in the distal conduit overlapping most of the Amplatzer™ Duct Occluder II (Fig. 1c). Control angiography revealed free insufficiency without extravasation of any contrast. RV-pressure had dropped to 46/0–9 mmHg at the end of the procedure.

**Fig. 2:**
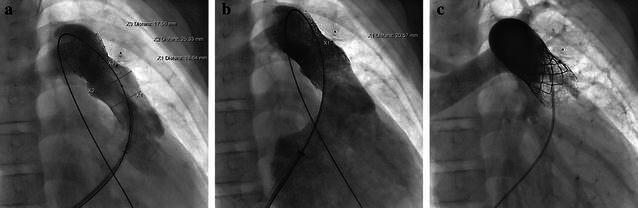
Re-catheterization revealing excluded pseudoaneurysm and stable pre-stent position (**a**). Well prepared landing-zone after implantation of two more pre-stents (**b**). Final result after percutaneous pulmonary valve implantation (**c**)

To minimize the risk of any device embolization, PPVI was scheduled 2 months later with the aim of ensuring the prestent’s stability while assuming some endothelial tissue ingrowth from the distal (native) pulmonary artery (Fig. 2). To reinforce the proximal landing zone, we implanted two more pre-stents (AS XXL 39 mm; Andramed, Reutlingen, Germany) before subsequently implanting the Melody valve via the 22 mm ensemble (Medtronic, Medtronic, Minneapolis, USA), followed by a final redilation with a 25/50 mm Cristal balloon achieving optimal fitting (Balt, Montmorency, France). The final angiogram showed an excellent outcome without any endoleak/aneurysmal formation. Final hemodynamic assessment demonstrated significant improvement and normal RV pressure. These findings remained stable during 3 year follow-up. There is no echocardiographic evidence of the valve’s mechanical dysfunction.

## Discussion

Pseudoaneurysms are a rare but potentially life-threatening complication after RVOT surgery. Factors implicated in their etiology are increased right ventricular pressure leading to mechanical strain at the suture line, the suture technique and materials, local infection, trauma, and significant pulmonary regurgitation (Levine et al. 1995; Sadiq et al. 1994). Considering the tendency for pseudoaneurysms to become progressively larger and risk rupturing, further treatment is needed (Antal et al. 2010; Levine et al. 1995). In most patients, the pseudoaneurysm can be resected under cardiopulmonary bypass on a beating heart. Surgery can be carried out safely with low mortality and morbidity, and is considered the mainstay treatment (Yeo et al. 1998; Murashita et al. 2002). On the other hand, as the pseudoaneurysm originates from the right ventricular outflow tract and lies just behind the sternum, there is the risk of bisecting during sternotomy. Thus, device occlusion may be considered an alternative in certain patients, particularly as percutaneous treatment of a dysfunctional right ventricular outflow tract has emerged as a recognized alternative as well, including techniques that may extend the use of PPVI beyond its current indications (Cools et al. 2015; Feltes et al. 2011; Holzer and Hijazi 2016; Jagia et al. 2011; Vaidyanathan et al. 2004). Another word of caution is needed when discussing pseudoaneurysms, however, as they actually carry a high risk of rupture. An underlying abscess/endocarditis must be considered a contraindication for any interventional approach.

In our case we decided on transcatheter two-stage approach, which included closing the pseudoaneurysm’s entrance with the Amplatzer device, followed by preparing the very compliant conduit for later PPVI. We were skeptical about the safety and durability of a long covered stent, as it might have carried a higher risk for stent migration and fracture via continuous mechanical (muscular) RVOT stress. We used non-covered stents with open-cell design for pre-stenting to provide sufficient anchorage in the retention zone while avoiding possible migration during or after valved stent implantation. The final valve implantation proceeded uneventfully. To optimize the result by flaring the stent-edges, we redilated 2–3 mm with an oversized balloon, which is usually well tolerated to this extent due to the Melody valve’s coaptation zone (Cheatham et al. 2013). Finally, we obtained a satisfactory result with adult-sized valve-diameters, thereby preserving the option for redo-intervention (valve-in-valve procedure) as a long-term solution.

## Conclusion

Percutaneous-transvenous exclusion of a large RVOT pseudoaneurysm, followed by PPVI in a very compliant RV-PA homograft succeeded, thus reducing both the risk for sudden rupture and the number of cardiac surgeries in this patient´s lifetime after a Rastelli procedure in early infancy.

### Additional files

**Additional file 1: Video S1.** Large pseudoaneurysm depicted at the level of the proximal homograft with its short neck and ostium.

**Additional file 2: Figure S1.** Relationship between the pseudoaneurysm and the inner sternal table to highlight the risk of bisecting during sternotomy.

**Additional file 3: Video S2.** Test-ballooning revealed only a little notching while ballooning the distal conduit.

## Abbreviations

RVOT: pseudoaneurysm of the right ventricular outflow tract
RV-PA: right ventricular-to-pulmonary artery
PPVI: percutaneous pulmonary valve implantation
RV: right ventricular

## References

[CR1] Antal AD, Cikirikcioglu M, Myers PO, Didier D, Kalangos A (2010). Respiratory distress after surgery of RVOT pathologies: a word of caution on pseudoaneurysm development. Thorac Cardiovasc Surg.

[CR2] Cheatham SL, Holzer RJ, Chisolm JL, Cheatham JP (2013). The Medtronic Melody(R) transcatheter pulmonary valve implanted at 24-mm diameter—it works. Catheter Cardiovasc Interv.

[CR3] Cools B, Brown SC, Heying R (2015). Percutaneous pulmonary valve implantation for free pulmonary regurgitation following conduit-free surgery of the right ventricular outflow tract. Int J Cardiol.

[CR4] Feltes TF, Bacha E, Beekman RH (2011). Indications for cardiac catheterization and intervention in pediatric cardiac disease: a scientific statement from the American Heart Association. Circulation.

[CR5] Holzer RJ, Hijazi ZM (2016). Transcatheter pulmonary valve replacement: state of the art. Catheter Cardiovasc Interv.

[CR6] Jagia P, Sharma S, Juneja R, Guleria R (2011). Transcatheter treatment of pulmonary artery pseudoaneurysm using a PDA closure device. Diagn Interv Radiol.

[CR7] Levine JC, Mayer JE, Keane JF, Spevak PJ, Sanders SP (1995). Anastomotic pseudoaneurysm of the ventricle after homograft placement in children. Ann Thorac Surg.

[CR8] Masri SI, Majdalani MN, Bitar FF (2014). Novel percutaneous femoral arterial-transthoracic approach for closure of ascending aortic pseudoaneurysm with a septal occluder device in a child. Cardiol Young.

[CR9] Murashita T, Hatta E, Imamura M, Yasuda K (2002). Giant pseudoaneurysm of the right ventricular outflow tract after repair of truncus arteriosus: evaluation by MR imaging and surgical approach. Eur J Cardiothorac Surg.

[CR10] Sadiq M, Fenton AC, Firmin RK (1994). False aneurysm of the right ventricular outflow tract after total correction of tetralogy of Fallot: diagnosis by echocardiography and successful repair by neck cannulation for cardiopulmonary bypass. Br Heart J.

[CR11] Vaidyanathan B, Kannan BR, Kumar RK (2004). Images in cardiovascular medicine. Catheter closure of pseudoaneurysm of the main pulmonary artery. Circulation.

[CR12] Yeo TC, Malouf JF, Oh JK, Seward JB (1998). Clinical profile and outcome in 52 patients with cardiac pseudoaneurysm. Ann Intern Med.

