# Supplementary figures and images for: Dysfunctional pulmonary artery conduit and co-existing large pseudoaneurysm: well-suited for a percutaneous approach with the Melody valve?
Source: Springerplus. 2016 Sep 15;5(1):1575. doi: 10.1186/s40064-016-3273-3 (PMC5025400; doi:10.1186/s40064-016-3273-3)

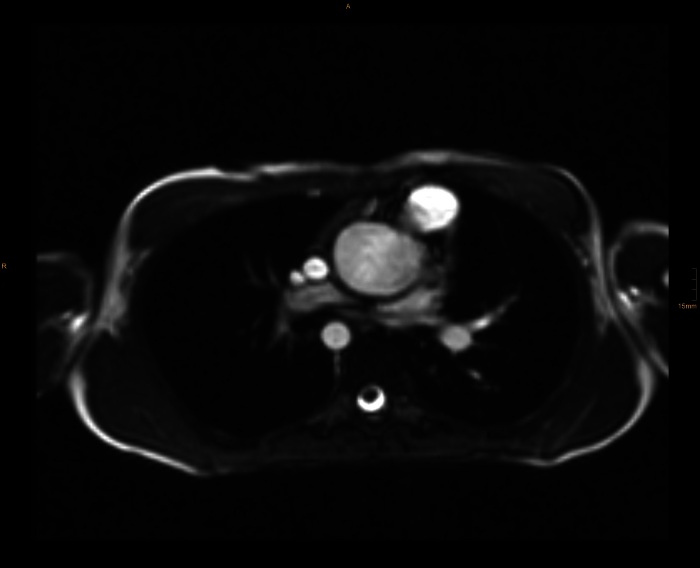

Supplement: Supplementary file 2 — Additional file 2: Figure S1. Relationship between the pseudoaneurysm and the inner sternal table to highlight the risk of bisecting during sternotomy. [file 40064_2016_3273_MOESM2_ESM.jpg]
